# An Optical Fiber Lateral Displacement Measurement Method and Experiments Based on Reflective Grating Panel

**DOI:** 10.3390/s16060808

**Published:** 2016-06-02

**Authors:** Yuhe Li, Kaisen Guan, Zhaohui Hu, Yanxiang Chen

**Affiliations:** Department of Precision Instruments, Tsinghua University, State Key Laboratory of Precision Measurement Technology and Instruments, Beijing 100084, China; gks13@mails.tsinghua.edu.cn (K.G.); huzhaohui470@126.com (Z.H.); chen-yx11@mails.tsinghua.edu.cn (Y.C.)

**Keywords:** fiber optic sensing, lateral displacement measurement, reflective grating panel

## Abstract

An optical fiber sensing method based on a reflective grating panel is demonstrated for lateral displacement measurement. The reflective panel is a homemade grating with a periodic variation of its refractive index, which is used to modulate the reflected light intensity. The system structure and operation principle are illustrated in detail. The intensity calculation and simulation of the optical path are carried out to theoretically analyze the measurement performance. A distinctive fiber optic grating ruler with a special fiber optic measuring probe and reflective grating panel is set up. Experiments with different grating pitches are conducted, and long-distance measurements are executed to accomplish the functions of counting optical signals, subdivision, and discerning direction. Experimental results show that the proposed measurement method can be used to detect lateral displacement, especially for applications in working environments with high temperatures.

## 1. Introduction

Displacement sensors and measurement techniques have been widely applied in many fields, including advanced fabrication machines, health monitoring devices, and medicine [1,2,3]. Capacitive sensors could provide excellent linearity, resolution, and bandwidth in short-range applications [4]. A sensing method with scanning probe microscopy (SPM) has become an indispensible tool for topographical measurement with nano- and sub-nanometer resolution [5], but usually the single scanning distance is of the order of 150 × 150 µm, and the scanning speed is also a limitation. Eddy current sensors have a large range with linearity error of less than ±3% [6,7], but they are not widely used because of the temperature sensitivity. Both linear variable displacement transformers [8] and linear encoders can supply nanoscale resolution with a large measurement range [9,10], however, they are unreliable in electromagnetic interference environments. Fiber optic sensors (FOS) have attracted much attention of researchers over the past few decades due to some innovative characteristics, such as high bandwidth, low loss, and can work under harsh environmental conditions compared to traditional sensors [11].

Fiber optic displacement sensors (FODS) are playing a more important role in the area of displacement measurement. Fiber Bragg grating-based sensors have become most widely known in the market due to their multiple functions [12,13,14,15,16,17]. Including interferometric sensors and intensity modulated sensors, many kinds of FODS have been presented in recent years [18,19,20,21,22,23,24,25,26,27,28,29]. The interferometric sensors could realize high sensitivity and accuracy, but they are usually complicated and of higher cost to set up wavelength shift interrogation systems [19]. The intensity-modulated sensors, by contrast, are simple and low cost [20,21,22,23,24,25,26,27]. A transmissive fiber lens double-fiber design is proposed to achieve displacement measurement [20]. A seond transmissive one could realize a measurement with an accuracy of 6 µm over a range of 3000 µm [21]. However, in the transmissive fiber sensor, the emitting fibers and receiving fibers are, respectively, arranged on two sides of the measuring probe, which usually occupies too much room [22]. Reflective fiber optic displacement sensors (RFODS) could simplify their structure by fixing the fibers on one side of the probe [23,24]. In addition, by adopting different reflective targets, RFODS show many advanced characteristics and could measure different measurands [28,29,30,31,32]. Although RFODS have high accuracy and simple structure, small measurement ranges, to some degree, limit their practical applications [33].

In this study, we propose a novel type of optical fiber measurement method based on a reflective grating panel for lateral displacement. The signal subdivision model with the tangent and cotangent functions is given, and error analysis and processing are studied. Based on the relationship between displacement and received optical power the phase difference is adopted in order to realize discerning direction for displacement measurement, and the center distance of two receiving fibers is derived theoretically to ensure the orthogonal phase between two receiving fibers. A distinctive fiber optic grating ruler is set up to measure dispalcement, which includes a special fiber optic measuring probe and reflective grating panel.

In this paper, an optical fiber measurement method for lateral displacement is presented, and a reflective grating panel is fabricated. The measurement method and system for displacement measurement is detailed. The simulation calculation of the optical path and resolution are carred out, and signal analysis and processing follows. Finally, some experiments including the application test in a high-temperature working environment, are presented to verify the proposed measurement method and the fiber optic grating ruler.

## 2. Measurement Method and System for Lateral Displacement

### 2.1. Working Principle

**Figure 1:**
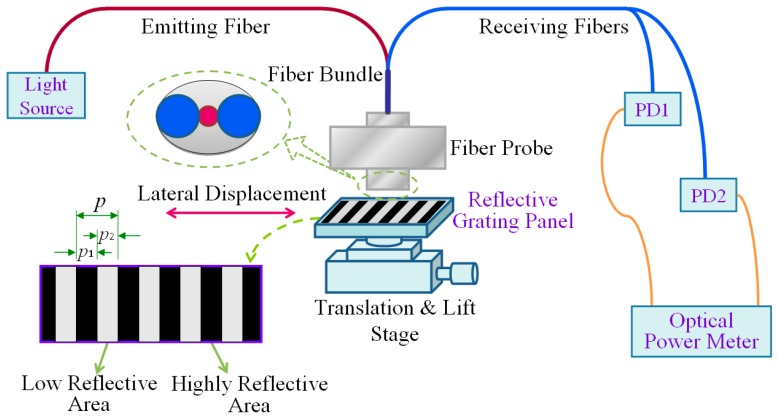
Displacement measurement system.

The displacement measurement system is composed of a light source, a sensor probe, a reflective grating panel (RGP), photodetectors (PD), and an optical power meter, as shown in Figure 1.

The RGP, which comprises highly-reflective areas and low reflective areas, is attached to the measurand. An emitting fiber and two receiving fibers make up the fiber bundle. The working process of the measurement system can be summarized as follows. The RGP moves with the measurand and the emitted light from the fiber is modulated by the moving RGP. When the light irradiates on the highly reflective area, the reflected light intensity is high, and *vice versa*. Through two receiving fibers, the modulated light transmits into PDs, whose signals enter the optical power meter. The reflected signals vary like sinusoidal functions, and they would vary one period when the RGP moves one grating pitch [33]. Therefore, the displacement measurement can be achieved by counting the sinusoidal signals. In the measurement system, the two receiving fibers are arranged at a specific location, so that the PDs could obtain two light signals *S*_1_ and *S*_2_ with a phase difference of 90°, which can be expressed as:

(1) $$S_{1}=\sin\left(\frac{2\pi \cdot x\left(t\right)}{p}\right)$$

(2) $$S_{2}=\cos\left(\frac{2\pi \cdot x\left(t\right)}{p}\right)$$

where *x*(*t*) is the measurand displacement, and *p* is the grating pitch. The displacement *x*(*t*) could be inferred as follows:

(3) $$x\left(t\right)=\frac{p}{2\pi }\arctan\left(\frac{S_{1}}{S_{2}}\right)$$

The signal processing methods adopted in the measurement system are similar to that used for traditional grating signals [34], such as counting, subdivision, and discerning direction.

### 2.2. Preliminary Theoretical Derivation

**Figure 2:**
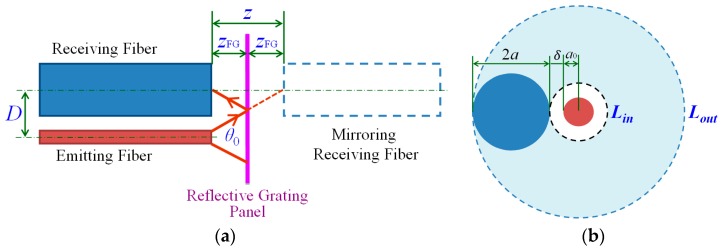
Schematic diagram of measurement method. (**a**) Layout of measurement units; and (**b**) spatial relations and dimension parameters.

The fiber-grating distance *Z*_FG_ between the fiber end and the RGP has influence on the measurement performance, as shown in Figure 2. The decreased *Z*_FG_ will result in a smaller spot size of fiber, which is related with the measurement resolution to some degree. When *Z*_FG_ gets larger than 0, the optical power of the receiving fiber will become higher, and then decrease gradually [23], which would have significant influence on the measuring sensitivity. Therefore, it is necessary to make an analysis about the relationship between *Z*_FG_ and the optical field intensity at the fiber end. On the basis of the intensity distribution of the output optical field formed by the source fiber end [35], the optical field intensity distribution function at the multimode fiber end is calculated by the following formula:

(4) $$I\left(r,z\right)=\frac{I_{0}}{a_{0}^{2}{\left[1+0.146{\left(z/a_{0}\right)}^{1.5}\tan\theta_{0}\right]}^{2}}\exp\left\{-\frac{r^{2}}{a_{0}^{2}{\left[1+0.146{\left(z/a_{0}\right)}^{1.5}\tan\theta_{0}\right]}^{2}}\right\}$$

where *I*_0_ is the intensity of the light source, *a*_0_ is the radius of the emitting fiber, *θ*_0_ is the fiber maximum exit angle, which is equivalent to the acceptance angle and can be determined by the numerical aperture (NA), *θ*_0_ = arctan(NA), *z* = 2*Z*_FG_ is the axial distance between the receiving fiber end and its mirroring, *r* is the radial distance from the fiber center, and *I*(*r*,*z*) is the light field intensity. The parameters of the emitting fiber are: *a*_0_ = 52.5 µm, tan*θ*_0_ = 0.22; then, by solving Equation (4), the following equation can be obtained:

(5) $$I\left(r,z\right)=\frac{I_{0}}{{\left(52.5+0.00445\times z^{1.5}\right)}^{2}}\exp\left\{-\frac{r^{2}}{{\left(52.5+0.00445\times z^{1.5}\right)}^{2}}\right\}$$

As shown in Figure 2, the emitting and receiving fibers are placed closely and parallelly, and the fiber ends are aligned as much as possible. The gray ring *L*_in_*L*_out_ is the emitted optical field. It can be known that the optical power of the receiving fiber is proportional to the light flux *B_Z_* in the area between the rings *L_in_* and *L_out_*. It can be expressed as:

(6) $$B_{z}=\int_{a_{0}+\delta }^{a_{0}+\delta +2a}I\left(r,z\right)dr=\int_{137.5}^{1137.5}I\left(r,z\right)dr=\frac{I_{0}\sqrt{\pi }}{105+0.0089\times z^{1.5}}\cdot \text{erf}\left(\frac{r}{52.5+0.00445\times z^{1.5}}\right)|\begin{array}{c} 1137.5\\ 137.5 \end{array}$$

where *a* = 500 µm is the radius of the receiving fibers, and *δ* = 85 µm is the interval between the emitting fiber and receiving fiber. It can be known from the numerical solution obtained by MATLAB software (MathWorks Inc., Natick, MA, USA) that *B_Z_* reaches a maximum value at the point of *Z* = 1292 µm. Therefore, when the fiber-grating distance *Z*_FG_ = 646 µm, the optical power of the receiving fiber will reach its maximum value.

## 3. Simulation Calculation

### 3.1. Optical Modeling

**Figure 3:**
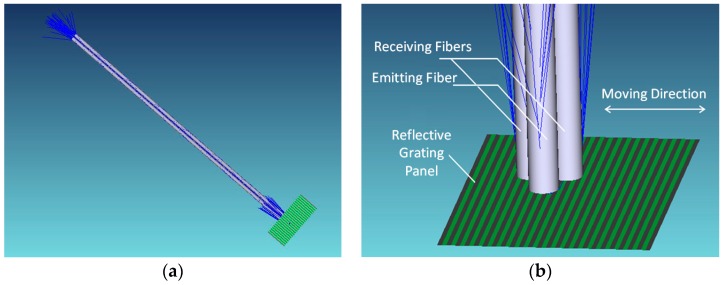
Simulation model of fibers and the RGP. (**a**) Overall view; and (**b**) partial enlarged view.

Simulation is conducted by using the optical design software, ZEMAX software (Radiant Zemax, LLC, Redmond, WA, USA). As shown in Figure 3, the simulation model consists of one LED light source, one emitting fiber, two receiving fibers and a RGP. The emitting fiber core diameter is 105 µm, and its numerical aperture, NA_1_ = 0.236. The receiving fiber core diameter 2*a* = 1000 µm, and NA_2_ = 0.467. The RGP grating pitch *p* is 300 µm. For simplicity, the reflectivity of high and low reflective areas were set to 1 and 0, respectively, and the highly-reflective area width *p*_1_ is equal to the low one *p*_2_.

### 3.2. Relationship between Fiber-Grating Distance and Received Optical Power

**Figure 4:**
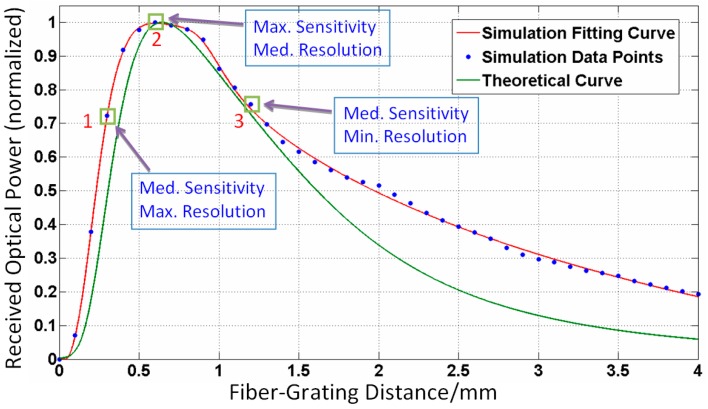
Comparisons between simulation fitting curve and theoretical curve.

The relationship between the fiber-grating distance *Z*_FG_ and the fiber received optical power has been derived above, and can now be verified through the following simulations. Assuming the emitting fiber is located on the center of the highly-reflective area, and *Z*_FG_ increases from 0 to 4 mm with a step of 0.1 mm, the average value of optical power from the two receiving fibers is obtained and normalized, then the curve of *Z*_FG_ and the received optical power can be fitted numerically, as shown in Figure 4. The comparison between fitting and theoretical curves indicates that both agree reasonably well. The fiber-grating distance *Z*_FG_ and the received optical power are of a non-linear relationship, and the maximum value of optical power is reached when *Z*_FG_ equals 600 µm. When the RGP moves with the measurand, the received optical power signal varies like a sinusoidal function. If the optical power gets larger, the peak-peak value of the signal becomes larger, accordingly. The measurement sensitivity is proportional to the optical power, theoretically. On the other hand, the light spot can be changed in size linearly with the parementer *Z*_FG_, and the identifiable grating pitch will vary accordingly. Therefore, there is an inverse relationship between the fiber-grating distance *Z*_FG_ and the measurement resolution on the whole. In summary, among the points 1, 2, and 3 in Figure 4, point 1 has the maximum resolution and medium sensitivity, point 2 has medium resolution and maximum sensitivity, and point 3 has the minimum resolution and medium sensitivity. To improve resolution and sensitivity, we choose the *Z*_FG_ of point 1 in the remaining simulations and experiments.

### 3.3. Relationship between Lateral Displacement and Received Optical Power

**Figure 5:**
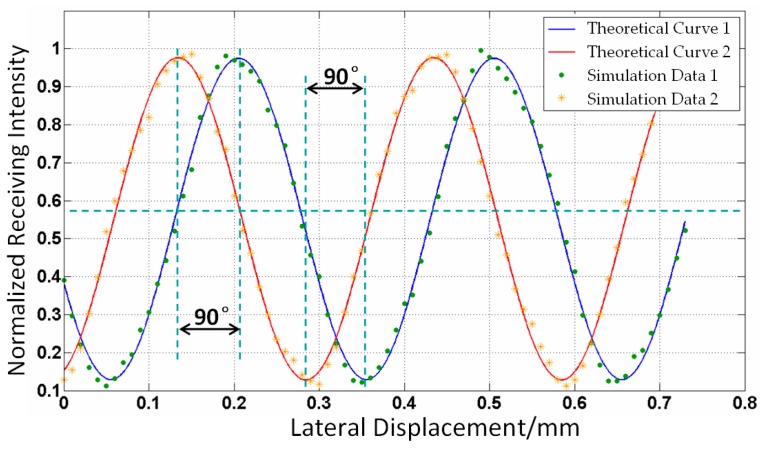
Simulation on relationship between received optical power and lateral displacement.

The simulation on the relationship between the lateral displacement and received optical power is conducted with the fiber-grating distance *Z*_FG_ = 0.3 mm, the pitch of RGP *p*_1_ = *p*_2_ = 0.5*p* = 150 µm. In order to get a phase difference of 90° between the two receiving fiber signals, the center distance of two receiving fibers *D* as shown in Figure 2, should be approximately equal to (*k* + 1/4)*p*; here, *k* is an integer. Due to space limitations, the center distance *D* takes the value 1275 µm (namely, *k* = 4). The center of a low reflective is set as a starting point. The two received optical power signals are, respectively, obtained and normalized, and the curve of lateral displacement and optical power can be fitted, as shown in Figure 5. The optical power signals vary like a sinusoid, with the cycle being the same as the pitch of RGP, 300 µm. From Figure 5, the signals from two receiving fibers have a phase difference of 90°, which can be used to realize the subdivision and discerning direction in order to improve measurement system performance.

## 4. Signal Subdivision and Error Analysis

**Figure 6:**
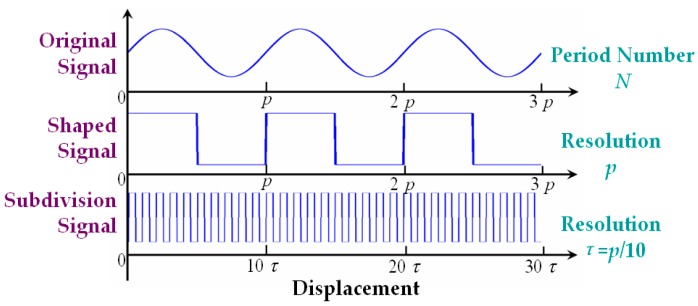
Schematic diagram of signal subdivision in displacement measurement.

**Figure 7:**
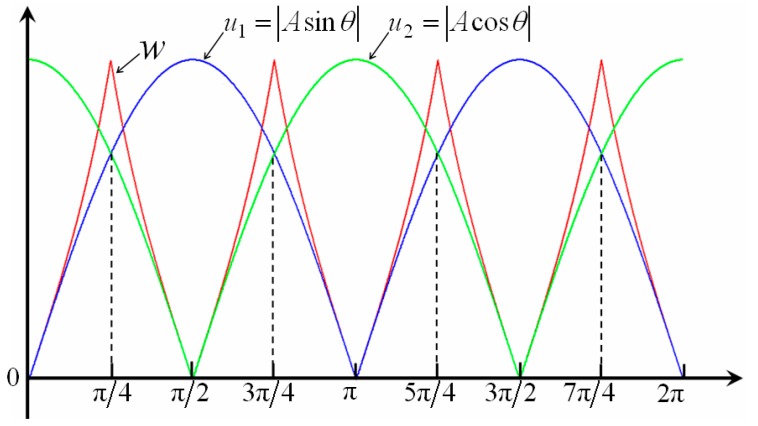
Ideal subdivision curve based on tangent and cotangent functions.

When the measured object moves, the reflecting grating and the optical fiber probe start relative motion at the same time, and the receiving optical fiber will receive a periodic signal modulated by the reflecting grating. As shown in Figure 6, the measured displacement can be obtained by the computational expression *x* = *N*·*p* = 3*p*, with the period number *N* = 3 and the resolution *p* (namely the known grating pitch). Assuming that the subdivision number is 10, then the resolution increase of up to 10 times can be achieved, and the displacement *x* = 30*τ*. Here the tangent and cotangent functions were used to realize signal subdivision for the modulated intensity from the RGP, which can be expressed as Equation (7) and shown in Figure 7.

(7) $$w=\{\begin{array}{c} \frac{\left|u_{1}\right|}{\left|u_{2}\right|}=\frac{\left|A\sin\theta \right|}{\left|A\cos\theta \right|}=\left|\tan\theta \right|\begin{array}{cc} , & \left|u_{1}\right|\leq \left|u_{2}\right| \end{array}\\ \frac{\left|u_{2}\right|}{\left|u_{1}\right|}=\frac{\left|A\cos\theta \right|}{\left|A\sin\theta \right|}=\left|\cot\theta \right|\begin{array}{cc} , & \left|u_{1}\right|>\left|u_{2}\right| \end{array} \end{array}$$

**Figure 8:**
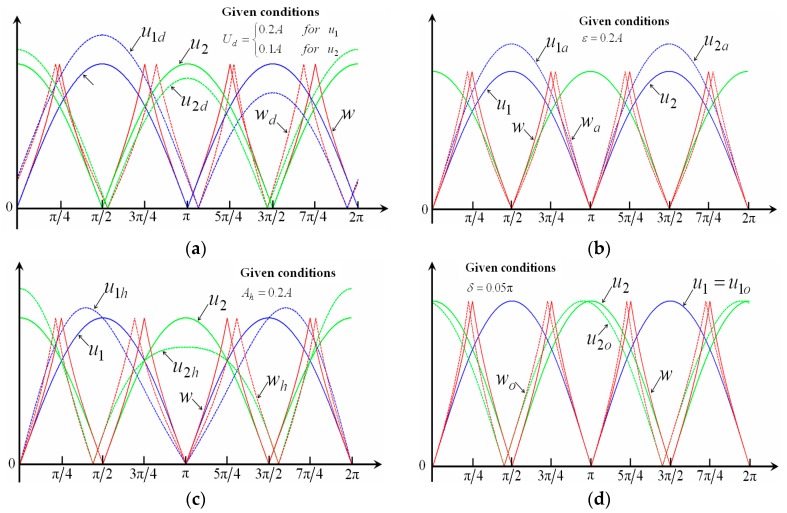
Error analysis of subdivision signals. (**a**) Error from the DC signal; (**b**) error from the signal amplitude variation; (**c**) error from the harmonic component; and (**d**) error from the non-orthogonal phase.

**Figure 9:**
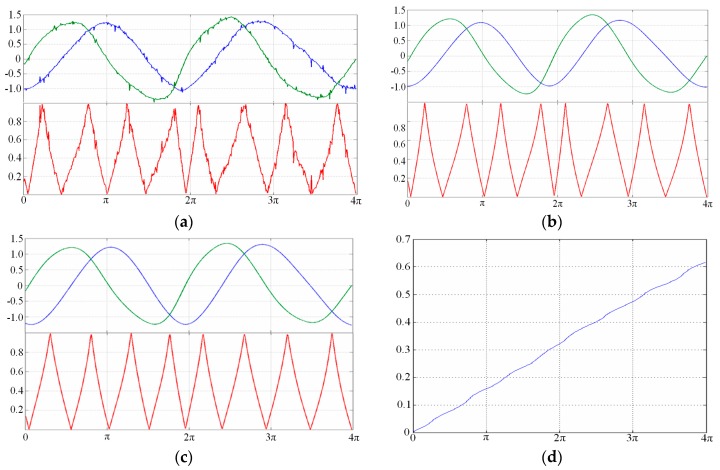
Signal subdivision processings. (**a**) Original signal (above) and tangent subdivision signal (below); (**b**) filtered signal by FIR; (**c**) signal by noise processing; and (**d**) displacement measurements with de-noising process.

Errors analysis of subdivision signals for displacement measurement is carried out for the following aspects: (a) the DC signal error *U_d_*; (b) the amplitude variation coefficient ε; (c) the harmonic component *A_h_*; and (d) the non-orthogonal phase error *δ*. For example, the change in the fiber-grating distance discussed above may cause the amplitude variation to some degree. The subdivision curves and signal subdivision processing are given in Figure 8 and Figure 9, and the error analysis shown in Table 1 [36].

## 5. Experimental Results and Discussion

### 5.1. Self-Made Fiber Optic Grating Ruler and Signal Processing

**Figure 10:**
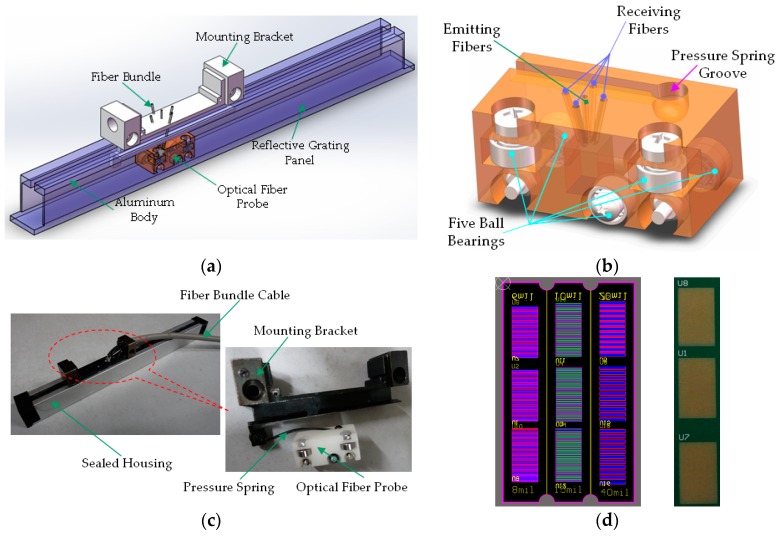
Fiber optic grating ruler. (**a**) Overall composition chart; (**b**) measuring probe; (**c**) overall ruler appearance and measuring probe; and (**d**) engineering drawings (left) and physical appearance (right) of RGP.

A fiber optic grating ruler was set up based on the homemade RGP, as shown in Figure 10. The RGP is made up of a printed circuit board (PCB), using gilded and striped pads as high reflective areas, the the substrate for low reflective areas. The measuring probe, including emitting and receiving fibers, spring groove, and guiding ball bearings, can move laterally above the RGP.

### 5.2. Displacement Measurement Experiments

**Figure 11:**
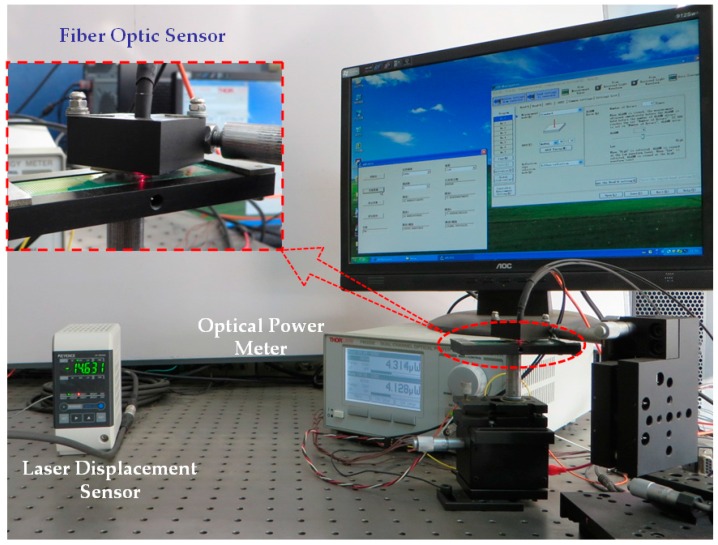
Experimental platform for lateral displacement measurement.

**Figure 12:**
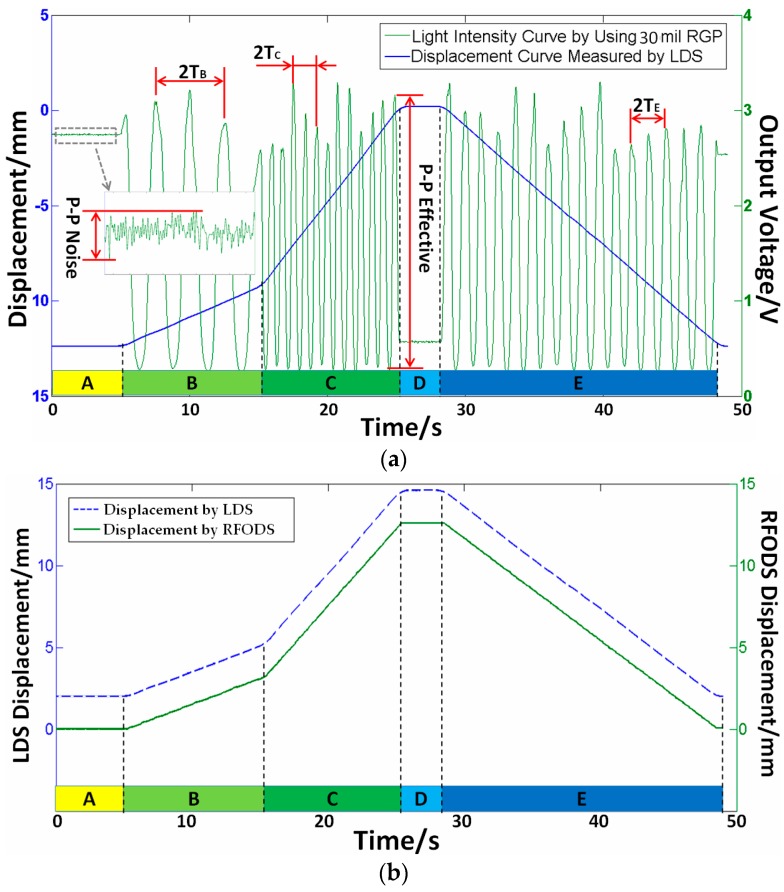
Comparisons between proposed RFODS (RGP pitch = 30 mil) and LDS. (**a**) Output voltage verse displacement; and (**b**) displacement from the RFODS and LDS.

To verify the effectiveness of the proposed method, an experimental platform was set up, as shown in Figure 11. Some system parameters, such as the signal to noise ratio (SNR), sensitivity, and resolution, have close relationship to the grating parameters. For comparison, three kinds of RGPs with different grating pitches of 12 mil (304.8 µm), 20 mil (508 µm), and 30 mil (762 µm), were fabricated. In order to simplify the analysis, the highly-reflective area width *p*_1_ is set equal to *p*_2_. A multimode glass fiber is adopted as the emitting fiber (M15L02, Thorlabs, Newton, NJ, USA), and plastic fibers as the receiving fibers (HFBR-RNS001Z, AVAGO, San Jose, CA, USA). A fiber-coupled high-power LED is selected as the light source (M660F1, Thorlabs, central wavelength 660 nm, FWHM 20 nm). A dual-channel power meter is used (PM320E, Thorlabs) and, correspondingly, two optical power sensors are adopted (S150C, Thorlabs). In the process of the experiment, the measurand moves according to following five stages: A-keep static for 5 s; B-move forward for 3.125 mm at a speed of 312.5 µm/s; C-move forward for 9.375 mm at a speed of 937.5 µm/s; D-keep static for 3 s; E-move backward for 12.5 mm at a speed of 625 µm/s. After the above stages, the measured object returns back to its initial position with the total movement of 25 mm. Generally, there are some measurement error factors, such as transmission clearance and losing steps of the stepper motor; therefore, the above-mentioned motion processes are monitored by a laser displacement sensor (LDS, LK-G400, LK-G3001, KEYENCE, Osaka, Japan) synchronously. The comparative data by the RFODS (single channel) and LDS are shown in Figure 12.

Figure 12 indicates that the output voltage matches the displacement data well. The velocity of the measured object can be obtained by processing either the displacement data from LDS or the output voltage data from RFODS. The relative measurement error was computed, for example, in stage B, where the velocities from the LDS and RFODS are 314.4 µm/s and 313.7 µm/s, respectively, and the relative error is about −0.223%. The measurement errors comparison under different pitches and different stages are listed in Table 2.

**Figure 13:**
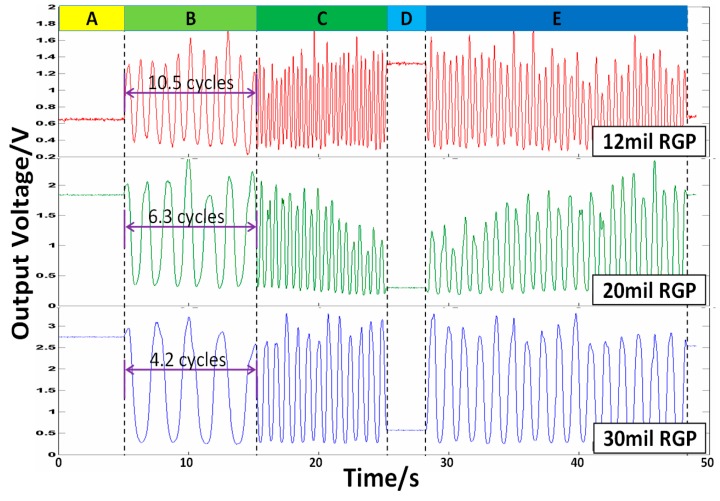
Comparisons of the output voltages under different RGP pitches.

The whole output from RFODS under different pitches are given in Figure 13, which shows that the signal cycle changes with the grating pitch. For example, the actual cycles in stage B are 10.5, 6.3, and 4.2 cycles for RGP pitches of 12 mil, 20 mil, and 30 mil, respectively, and their relative errors are about 2.4%. However, smaller pitch is easier to be influenced by the machining process. As shown in Figure 13, in stages A and D, the measured object remains static, so the output voltage can be treated theoretically as noise signals of the RFODS. Then, the signal to noise ratios under different pitches can be calculated and listed in Table 3. We can see that the pitch is generally inversely related to the SNR.

**Figure 14:**
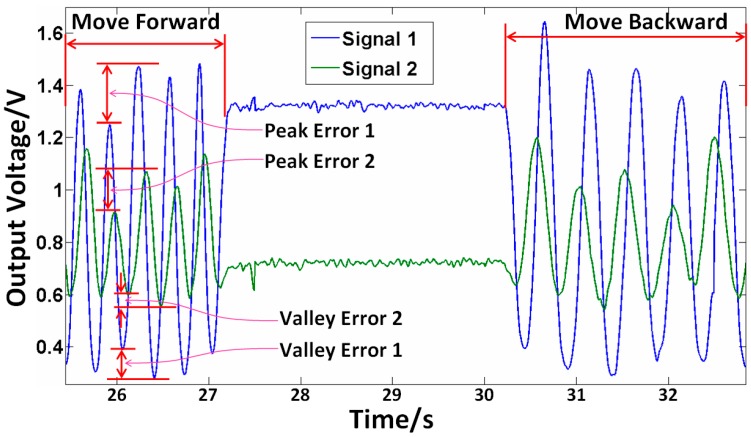
Two receiving fiber signals with phase difference.

In addition, as shown in Figure 14, the two receiving fibers collect two light signals, whose phase difference is approximately equal to 90°. Based on phase difference, the measurement resolution could be improved to about 0.76 µm with 400-fold subdivision. When the RGP moves forward, the phase of signal 1 is behind that of signal 2, and *vice versa*, then the function of discerning direction can be realized practically. Moreover, the exit peak and valley errors mainly caused by the reflectivity inhomogeneity of the RGP, which would lead to the reduction of measurement accuracy [34]. Through high-precision fabrication processes and assurance measures, the measurement accuracy could be improved accordingly.

### 5.3. Large Range Measurement Experiments

It can be known from the system structure and operating principle that the measurement range is related to the length of the RGP, and a large range measurement could be obtained with a long RGP. In these experiments, RGP length takes the value of 300 mm, and the grating pitch distance of 12 mil. The lengths of the emitting fiber and receiving fibers are both 40 m.

**Figure 15:**
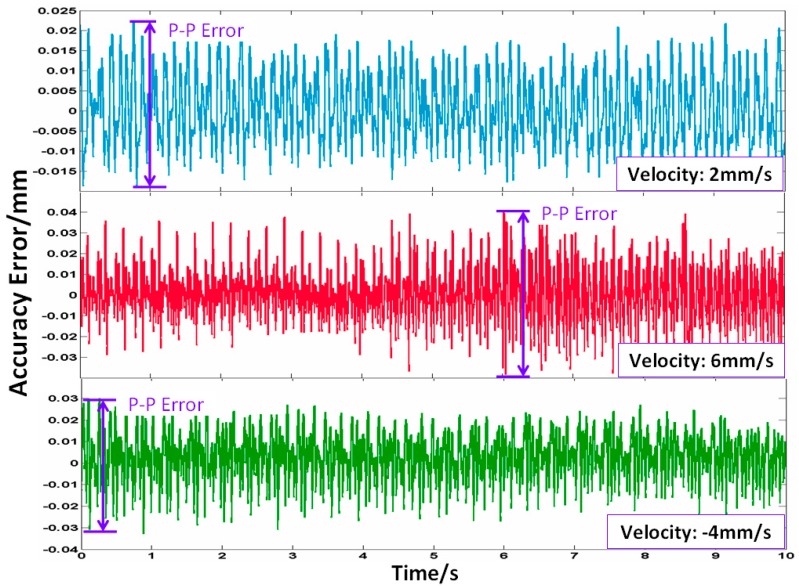
Accuracy error between the proposed RFODS and LDS under different velocities.

In order to investigate the system accuracy, the measured object moves by the following stages: (a)-move forward for 50 mm at a speed of 2 mm/s; (b)-move forward for 150 mm at a speed of 6 mm/s; and (c)-move backward for 200 mm at a speed of 4 mm/s. The total measurement range is 400 mm. Figure 15 has shown the accuracy errors between the proposed RFODS and commercial LDS under different velocities from the initial movement time to 10 s in each stage. The peak-peak accuracy errors are ±0.0205 mm, ±0.0393 mm, ±0.0303 mm in (a), (b), (c) stages, respectively. It is obvious that the measurement system could maintain its accuracy well over a large measurement range.

### 5.4. Temperature Test

**Figure 16:**
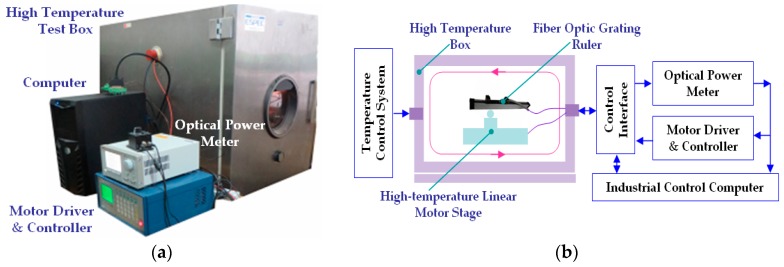
Experimental system for temperature test. (**a**) Overall system appearance; and (**b**) system architecture schematic.

Due to the capability of remote measurement by optical fiber, only the fiber probe is needed to be put near the measurand; the other components could be placed in a favorable environment away from the measurand. Therefore, the proposed RFODS has the capability to work in some harsh conditions, such as high to low temperature. To obtain system performance in high to low temperature working environments, an incubator is used to set up the experimental platform. As shown in Figure 16, the measurand, fiber probe, RGP, and stages are placed inside the incubator, while the other modules are put outside. In the experimental process, the incubator temperature change from −20 to 80 °C by every 10 °C. At each temperature, the measurand moves forward for 20 mm at a speed of 625 μm/s three times. At the same time the light signals are acquired by the device outside the incubator. The movement velocities under different temperatures can be obtained, as shown in Table 4, where *V*_1_, *V*_2_, and *V*_3_ are the velocities for each time, respectively. The average velocity of three times at 20 °C is treated as the nominal value. Accordingly, the relative errors *α*_1_, *α*_2_, and *α*_3_ could also be computed and shown in Table 4.

It can be seen that the proposed RFODS works well in the temperature range from −20 to 80 °C, and the relative error is less than ±0.5%. When the temperature is higher than 80 °C, the system performance is influenced by softening and deformation of the fibers. With glass or special optical fibers, the measurement system has the potential to work at higher temperature.

## 6. Conclusions

In this paper, we demonstrate an optical fiber measurement method and experimental system for lateral displacement based on a reflective grating panel. Through theoretical derivation, the optimized distance between the emitting fiber ends and the reflective grating panel is obtained. By means of modeling and simulation, the efficiency parameters are verified, and the relationship between the displacement and the received optical power is summarized. Experiments under different grating pitches indicate that the proposed method can achieve a long-distance measurement, especially for applications in harsh working environments with high temperatures. Moreover, utilizing the two receiving fiber signals with phase difference, discerning direction and subdivision could be realized. If larger multiples of subdivision are applied, and the reflective grating panel fabricated precisely, there is potential to reduce system uncertainties and greatly improve the measurement accuracy.

## Figures and Tables

**Table 1:** Error analysis for signal subdivision (subdivision 100).

Error Type	Accumulation Interval	Quantitative Requirements	Ensuring Measures for Precision Subdivision	Parameter Index	
				Before Noise Processing	After Noise Processing
DC signal	π/4	*U_d_* < 3.68%	Original signals are subtracted from average value	*U_d_* = 0.1373*A*	*U_d_* = 0.0304*A*
Amplitudes variation	π/2	ε < ±11.8%	signal peak-to-peak amplitudes are used to correct the error	ε = 0.1884*A*	ε = 0.0086*A*
Harmonic component	π	*A_h_* < 0.038*A*	FIR low-pass filtering	*A_h_* = 0.0523*A*	*A_h_* = 0.0351*A*
Non-orthogonal phase	π	*δ* < 0.0133π	Calculation of phase difference based on the position of signal peak	*δ* = 0.0741π	*δ* = 0.0286π

**Table 2:** Comparison of measurement error between proposed method and LDS (1 mil = 25.4 µm).

Pitch	Stage 1 Stage 2 Stage 3	Velocity by LDS *V*_L_ (µm·s^−1^)	Velocity by RFODS *V*_R_ (µm·s^−1^)	Relative Measurement Error (*V*_R_ − *V*_L_)/*V*_L_
	Stage B	315.8	316.9	0.348%
12 mil	Stage C	936.0	937.8	0.192%
	Stage E	−626.9	−627.6	0.111%
	Stage B	315.6	315.9	0.095%
20 mil	Stage C	933.4	933.6	0.021%
	Stage E	−628.2	−628.9	0.111%
	Stage B	314.4	313.7	−0.223%
30 mil	Stage C	935.9	938.7	0.299%
	Stage E	−627.6	−625.6	−0.319%

**Table 3:** SNR with different grating pitches (1 mil = 25.4 µm).

Pitch (mil)	P-P of Noise Signal (V)	P-P of Effective Signal (V)	SNR
12	0.050	1.191	21.655
20	0.026	1.709	65.731
30	0.021	2.885	137.381

**Table 4:** Analysis of measurement error between proposed RFODS and LDS.

Temperature (°C)	*V*_1_ (μm/s)	*V*_2_ (μm/s)	*V*_3_ (μm/s)	*α*_1_	*α*_2_	*α*_3_
−20	622.2	621.9	621.8	−0.188%	−0.236%	−0.252%
−10	622.8	623.3	623.2	−0.091%	−0.011%	−0.027%
0	623.4	623.7	624.6	0.005%	0.053%	0.197%
10	623.8	622.7	623.4	0.069%	−0.107%	0.005%
20	623.7	622.7	623.7	0.053%	−0.107%	0.053%
30	624.3	624.0	624.3	0.149%	0.101%	0.149%
40	624.3	625.8	625.3	0.149%	0.390%	0.310%
50	625.5	626.3	625.4	0.342%	0.470%	0.326%
60	622.2	623.9	624.6	−0.188%	0.085%	0.197%
70	625.1	624.7	625.3	0.278%	0.213%	0.310%
80	624.5	625.5	625.3	0.181%	0.342%	0.310%
